# Platelet Proteome and Tumor Dormancy: Can Platelets Content Serve as Predictive Biomarkers for Exit of Tumors from Dormancy?

**DOI:** 10.3390/cancers2020842

**Published:** 2010-05-11

**Authors:** Nava Almog, Giannoula Lakka Klement

**Affiliations:** Center of Cancer Systems Biology, Caritas St. Elizabeth's Medical Center, Tufts University School of Medicine, Boston, MA, USA

**Keywords:** tumor dormancy, angiogenesis, platelets, early tumor detection

## Abstract

Although tumor dormancy is highly prevalent, the underling mechanisms are still mostly unknown. It is unclear which lesions will progress and become a disseminated cancer, and which will remain dormant and asymptomatic. Yet, an improved ability to predict progression would open the possibility of timely treatment and improvement in outcomes. We have recently described the ability of platelets to selectively uptake angiogenesis regulators very early in tumor growth, and proposed their use as an early marker of malignancy. In this review we will summarize current knowledge about these processes and will discuss the possibility of using platelet content to predict presence of occult tumors.

## 1. Introduction

Dormant tumors are highly prevalent in both normal subjects as well as in cancer patients. They are undetectable by most currently used imaging technologies. Dormant tumors are defined as microscopic, asymptomatic, histologically malignant lesions that remain occult for prolonged periods of time. While dormancy in primary tumors is best defined as the time between the carcinogenic event and the onset of progressive growth, it can also occur as “minimal residual disease”, occult leftovers from treated tumors or as non-growing micro-metastases. We have developed and characterized *in vivo* experimental models of human tumor dormancy in which the dormancy period is associated with impaired angiogenesis and the transition to rapid tumor growth phase is marked by intense neovascularization [1,2]. In these models, we described distinct molecular changes, which underlie the transition from dormancy to rapid tumor growth, and could be targeted for tumor growth modulation and therapy [3]. Because many of these tumor specific molecular changes are reflected in the protein profiles of circulating blood platelets [4,5], we suggested that platelets can be used as biomarkers of tumor growth, regression, recurrence or therapeutic response. 

Platelets are small corpuscles that circulate freely in intact vessels, but adhere readily to injured endothelium. In addition to their well-known role in thrombosis and hemostasis, platelets are sources of angiogenesis regulators necessary for the repair of injured tissues, and tumor growth. The angiogenesis regulating proteins are organized in separate compartments within platelets facilitating differential release of either stimulators or inhibitors of angiogenesis. Thus, depending on specific stimuli arising from the reciprocal interactions of platelets with the various cells within the local microenvironment, platelets can stimulate or inhibit the growth of a tumor. We recently reported that even microscopic human xenograft tumors in mice could affect the concentrations of angiogenesis related proteins in circulating platelets [4,5]. We termed this group of proteins “platelet angiogenesis proteome”, to emphasize their ability to modulate angiogenesis in health as well as disease. 

This review introduces the concept of a close interplay between the platelet angiogenesis proteome and the escape of tumors from dormancy. We surmise that an improved understanding about the manner through which tumors are induced to remain dormant would have important implications for cancer treatment and cancer screening, but, to date, the dormant phase of tumor growth is an unexplored therapeutic target. This is due to difficulties in detecting dormant tumor cells in a clinical setting, as well as difficulties with the interpretation of an incidental finding of an occult tumor. It is also unclear at present which of the tumors will remain dormant and for how long, and which will progress, hindering any consideration for early interventions. In this review we will explore the possibility of using circulating platelets content as predictive markers to identify the critical decision point at which dormant tumors acquire the necessary changes and gain the angiogenic capacity required to transit from dormancy into exponential tumor growth.

## 2. Tumor Dormancy

Cancer dormancy is a stage in tumor progression during which tumors are kept occult and asymptomatic for a prolonged period of time. It had been presumed that dormancy represents a pre-invasive state of cancer progression, as it can be one of the earliest stages in primary tumor development. However, the observation that micro-metastases in distant organs, and minimal residual disease left after surgical removal or treatment of primary tumors can also enter dormancy, had introduced the likelihood that dormancy is a result of host/tumor interaction within the tumor microenvironment. 

Microscopic dormant tumors are very common and are highly prevalent in otherwise healthy people [6,7,8,9,10,11]. They are a frequent observation in autopsies of subjects who died of other causes. Due to their small size and the absence of any associated systemic symptoms, most cases remain undetected [12,13]. Yet, the incidental finding of a small, asymptomatic tumor poses a difficult clinical question, as there are no tools to guide a treatment decision. With advances in diagnostic imaging and molecular biology, it is now becoming clear that such tumors can remain in this asymptomatic, dormant stage for very long periods of time. Because of the few tumors that eventually escape from dormancy and progress, we extend undue clinical surveillance, add further to patient anguish, and squander valuable resources. The discovery of ductal carcinoma *in situ* (DCIS) represents a specific example of the clinical dilemma posed by the lack of understanding about dormancy. These tumors can remain dormant indefinitely, but a select few will go on to progress to devastating disease. Should all women with DCIS be treated to prevent the possibility of progression? Should we, in order to prevent progression, risk over treating women whose tumors would have never progressed with very toxic therapies [14]? Is it possible that some of our interventions worsen the natural history of these dormant lesions [15,16]? A similar clinical dilemma is presented by the long periods of dormancy which occur after a successful initial treatment of breast cancer. The National Cancer Institute of Milan, Italy study, which included 1173 women who underwent mastectomy as single initial treatment for early stage breast cancer, indicated a bimodal pattern of cancer recurrence, with an early, rather sharp, dominant peak at about two years and a second broader peak at about five years with a decay that extended to 15 years. What are the factors influencing these prolonged periods of dormancy? 

The dormancy period depends on certain molecular and cellular mechanisms, which either actively halt tumor progression or are insufficient to enable tumor progression. These can be considered as decision points at which the equilibrium of dormant tumors is levied against a new host environment. If the balance of regulating factors is disturbed, the dormant tumor may be released from growth restraints, inducing exponential tumor mass expansion. Although dormant tumors are highly prevalent, very few of them progress to disseminated disease, suggesting that if changes at these decision points could be detected, escape from dormancy may be prevented. Unfortunately, very little is currently known about the mechanisms responsible for induction of tumor dormancy or about factors affecting escape from dormancy.

Clinical data clearly show the high prevalence of dormancy of both primary and secondary tumors [7,8,12,17,18]. Autopsy studies indicate that presence of occult and microscopic tumors in otherwise healthy people is much higher than previously estimated [6,8,12]. Cancer recurrence after therapy and long periods of remission is frequent. Dormancy of primary tumors can be evident also as minimal residual disease, which describes the presence of traces of tumor cells at site left after an apparently successful treatment of the original tumor. Cancer patients believed to have good prognosis can still develop metastasis or have re-growth of tumor at the primary site many years, or even decades, after treatment [12]. It is well accepted that tumors shed cells even at early stages in tumor development and that these disseminated tumor cells can remain dormant for prolonged periods of time. These disseminated cells, which can eventually emerge and initiate growth, regardless of presence of primary tumor, could explain metastatic relapse often seen in cancer patients following a surgical, radiation or chemotherapeutic intervention.

Although highly prevalent, tumor dormancy is currently one of the most neglected research areas in tumor biology. Until very recently, intervention with existing highly toxic therapeutic agents could not be justified in an otherwise asymptomatic patient, and there was little stimulus to study cancer dormancy. However, with the emergence of biological response modifiers and minimally toxic anti-cancer therapies, a newly found awareness has surfaced and new theories about the mechanisms that underlie this stage of tumor progression are emerging. Arrested angiogenesis is a well known mechanism of tumor dormancy [1,2,8,19,20,21,22,23,24,25,26,27], but additional biological processes could affect tumor dormancy as well. It is well documented that stimuli from microenvironment can induce a cell cycle arrest [17,28,29], which can result in cellular dormancy of tumor cells. Cellular dormancy can occur at the level of single ‘solitary’ tumor cells or in tumor foci. Quiescent solitary tumor cancer cells have many different characteristics from those of actively proliferating cancer cells in non-angiogenic dormant tumors. The immune system is also well known to play a role in tumor progression and several experimental models had suggested that the immune surveillance can induce tumor dormancy [30,31,32,33]. However, since many of the immune cells are known to express angiogenic factors and to be tightly coupled to angiogenesis processes, it is possible that several of the effects on tumor dormancy could be mediated by blocking or interfering with angiogenesis. Other mechanisms that had been suggested to regulate tumor dormancy are tumor metastases genes [34], diet [35], hormonal control and autophagy [36,37].

## 3. Induction of Angiogenesis as a Decision Point

All tumors depend on recruitment of functional blood vessels to support the growth of the tumor mass [38,39,40]. It had been shown in many experimental models that tumors unable to induce successful angiogenesis remain avascular and microscopic in size [1,2,21,23,26,41,42,43]. Moreover, prolonged dormancy associated with impaired angiogenesis has been reported in many tumor types [1,2,21,22,27,42,44,45,46,47,48]. In these experimental models, tumor cells fail to form progressively growing tumors, instead forming small lesions that remain asymptomatic at the site of injection. Tumor cells in avascular dormant tumors exhibit a high proliferation rate that is balanced by elevated apoptosis. The escape from dormancy is associated with a shift in the angiogenic balance of the tumors. This transition from the pre-vascular lesion to highly vascularized and progressively outgrowing tumor is referred to as the “angiogenic switch”. Therefore, non-angiogenic tumors will remain dormant until they acquire sufficient mutations or changes to induce the angiogenic switch and become vascularized rapidly growing tumors.

The association of tumor dormancy and arrested angiogenesis has been described in *in vivo* experimental models of human dormant breast cancer, glioblastoma, osteosarcoma, and liposarcoma [1,2]. In these models, human tumor cell lines injected into SCID mice generate small tumors that remain undetectable by gross examination for prolonged periods of time. The time for the switch depends on the tumor types. Cells taken from tumors that had switched and became angiogenic would form fast-growing and angiogenic tumors once re-injected into new mice. Therefore, a stable, genetic reprogramming occurs during their switch to the fast-growing phenotype. 

By comparing the molecular and cellular characteristics of cells that form dormant tumors to those of cells forming fast growing tumors, we found that both types of cells contained fully transformed cells, and had similar proliferation rates *in vitro* [2]. However, a significant difference was observed in tumor vasculature structure between dormant and fast growing tumors. Throughout the dormancy period, development of intra-tumoral vessels was impaired. The non-angiogenic dormant tumors contained small clusters of endothelial cells without lumens, suggesting that the process of tumor angiogenesis was incomplete. Moreover, microvessel density, as observed by CD31 staining, decreased in tumors throughout the dormancy period. Cells from non-angiogenic dormant tumors secreted relatively high levels of the potent angiogenesis inhibitor, thrombospondin [1,2]. Therefore, tumor dormancy was clearly associated with arrested angiogenesis and with inability to sustain or induce formation of functional blood vessels. 

There is significant support for the theory that dormancy is due to the inability of tumor cells to overcome certain intrinsic thresholds. We recently reported the molecular determinants of angiogenesis-related tumor dormancy in four tumor types: human breast carcinoma, glioblastoma, osteosarcoma and liposarcoma [3]. In this work, genome-wide transcriptional analysis was used to compare gene expression profiles of cells that form either dormant or fast growing tumors for each of the four tumor types. Genes that were differentially regulated and had the same expression pattern in all tumor types were then identified. These genes constituted the consensus molecular signature of dormant tumors and provided insights into the molecular determinants of human tumor dormancy [3]. By an analysis of enriched gene ontology processes, the study demonstrated that the most differentially regulated between the dormant and the fast growing tumor phenotypes was the angiogenesis pathway. This confirmed, on the transcriptional level, the important role of angiogenesis as a mechanistic basis for the phenotypic differences observed between dormant and fast growing tumors. Among the genes that were up-regulated in all dormant tumors, thrombospondin is one of the best characterized angiogenesis inhibitors. Maintenance of a high level of thrombospondin secretion is a critical component of tumor dormancy [1,2,49,50]. These results do not exclude, however, the involvement of additional pathways and mechanisms in cancer dormancy, but rather strengthens the importance of angiogenesis blockage in maintaining tumors in a harmless state.

The specific molecular players identified to be differentially expressed between dormant and fast-growing tumors further strengthen the critical role of angiogenesis induction in the transition to exponential tumor growth. This angiogenic switch is a local phenomenon mostly restricted to the immediate vicinity of the early growth. At least in the early stages of tumor expansion, the systemic levels of free angiogenesis regulators in plasma remain undetectable, making early discovery of this tumor transformation very difficult. However, our discovery that the localization of early tumor angiogenesis is facilitated by platelets and that platelet content of angiogenesis regulators is therefore altered very early in tumor growth has provided a new modality for early cancer detection. 

## 4. Platelets and Their Role in Tumor Biology

While the role of platelets in thrombosis and hemostasis is well established, it is not always appreciated that they fulfill an important function in tissue repair, maintenance of endothelium, vascular tone and tumor growth. This function extends beyond their ability to create a thrombus and provide an extracellular matrix along which new vasculature can grow and expand. We have shown recently that platelets actively sequester angiogenesis regulators in the context of tumors [4,5] and wounds [51]. This sequestration is active, as it happens against a concentration in plasma; and highly selective for angiogenesis regulators. While many proteins may be non-specifically taken up by platelets [52], the differential levels of these proteins between platelets and the corresponding plasma have only been described for angiogenesis regulators. We have also documented a tightly controlled organization of angiogenesis regulating proteins such as VEGF and endostatin within the platelet alpha granules and suggested that there may be a differential release of angiogenesis stimulators and inhibitors, depending on tissue specific proteases [53].

The changes in the delivery of angiogenesis regulators to the sites of active angiogenesis can occur by at least two different means. Changes in the levels of angiogenesis regulators may occur within the platelet, or the actual number of circulating platelets may change. Clinical experience supports both processes. The growth of some tumors, such as is the case of multiple myeloma or neuroblastoma, is associated with increased platelet counts. However, in most tumors the changes in tumor levels of angiogenesis regulators happen independently, and often in absence, of any changes in platelet count. This is an important realization, because platelets are an acute phase reactants and their numbers can change non-specifically with any inflammatory process. On the other hand, the changes in the content of proteins in platelets are a result of an active and very selective process, resulting in a very consistent, highly reproducible and specific effect not dependent on inflammation. The bioavailability of proteins in platelets is minimal both under physiological as well as pathological conditions [4,87].

**Figure 1:**
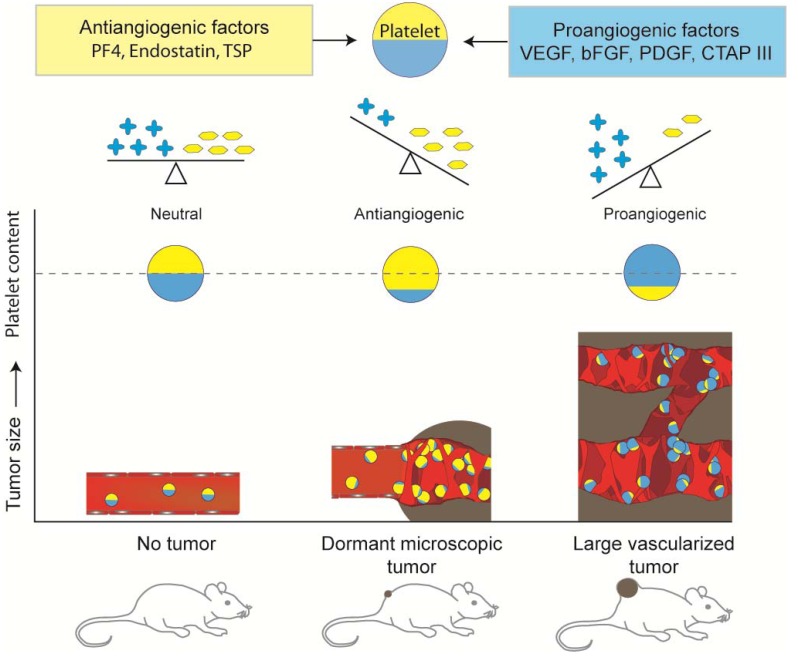
Schematic presentation of changes in the balance of angiogenic factors in circulating platelets, which reflect the presence of tumors. Presence of even a microscopic dormant tumor can induce an increase in level of antiangiogenic factors in platelets, while presence of large, fast growing and angiogenic tumor results in an increase of angiogenic factors [4,5]. It should be recognized that this overall decrease or increase in the respective angiogenesis phenotype represent a change in the overall angiogenic function and that platelets of dormant tumor bearing mice may have a significant amount of pro-angiogenic proteins that are balanced by sufficient numbers of inhibitors.

The finding that platelets contain proteins that regulate angiogenesis is not new [54,55,56,57], but it had not always been appreciated that the platelet concentrations of angiogenesis regulatory proteins, while relatively constant and stable under physiologic conditions, are modified by the presence of a malignant tumor growth [4,5]. We have shown that in the presence of even a microscopic (<1.0 mm) nonangiogenic tumor in a mouse, circulating platelets actively sequester angiogenesis regulatory proteins. The scheme in Figure 1 summarizes the present understanding by the authors of this balance. The uptake of angiogenesis regulatory proteins is discerning, as platelets do not take up other plasma proteins. For example, although albumin is present in plasma at much higher concentrations than VEGF, albumin levels in platelets do not differ in the presence or absence of tumors [5]. 

Before it was appreciated that the process of sequestration of angiogenesis regulators is an active phenomenon that happens against a concentration in plasma, and is highly selective for angiogenesis regulators, it was thought that the presence of both inhibitors and stimulators of angiogenesis in platelets was a result of nonspecific diffusion of these proteins into platelets from plasma. The selectivity of the process and the large difference between the concentration of the respective proteins in platelets and plasma suggest otherwise. Because of the highly selective nature of the process the levels of inhibitors and stimulators of angiogenesis in platelets, are more likely to be a result of well-orchestrated host response to the local tumor invasion. While we suggest in this manuscript that this balance may serve as a marker of tumor growth, it is also likely that this physiological relevance may be harnessed with therapeutic intent. 

The close interaction of platelets and the tumor microenvironment, and the ability of platelets to reflect the presence of a microscopic tumor suggested the possibility that the platelet content of angiogenesis regulators (the platelet proteome) may provide a tool for the detection of specific decision points along the transition from dormancy to exponential tumor growth. In our initial study, we employed high-throughput SELDI-ToF MS (Surface Enhanced Laser Desorption/Ionization—Time of Flight Mass Spectrometry), which permitted a rapid analysis of a large number of samples, in a highly efficient and reproducible manner [58,59], to analyze the protein content of platelets [4,5]. We did not restrict the analysis to candidate angiogenesis related proteins, but rather sought to identify all proteins differentially expressed in the platelets of mice bearing human tumor xenografts of either dormant or fast-growing clones, and the corresponding tumor-free controls. Interestingly, to date, all of the differentially expressed proteins we have identified in platelets from tumor bearing *vs*. non-tumor-bearing animals were angiogenesis regulators. Furthermore, the changes in platelet angiogenesis proteome reflected the presence of dormant, microscopic-sized tumors in mice months before these tumors could be detected by conventional methods and before the angiogenesis regulatory proteins could even be detected in plasma. The platelet concentrations of accumulated angiogenesis regulatory proteins continued to increase or remain significantly elevated for as long as a tumor was present (>100 days), despite the short life span of mouse platelets (~4–7 days).

Angiogenesis is a critical element of many physiologic processes such as wound healing, as well as pathologic processes such as tumor growth. In the former, an orchestrated up- and down-regulation of angiogenesis is necessary, in the latter, the continuous growth creates a moving tumoral margin, *i.e.*, “a wound that never heals” [60]. In both situations, the initial blood vessel sprout is driven locally by the release of pro-angiogenic factors such as VEGF and bFGF. In the case of wound-healing, the vessel is stabilized by another set of proteins such as PDGF, and the tissue re-organization results in an inhibition of angiogenesis by local release of antiangiogenic factors such as endostatin, PF4 and thrombospondin from platelets. 

This sequential release of angiogenesis regulators from platelets is enabled by a higher organization of angiogenesis regulating proteins within the platelet alpha granules [53]. As a consequence of this higher organization, the individual proteins within the distinct alpha-granule compartments undergo differential release of angiogenesis stimulators and inhibitors from platelets depending on the interaction of platelets with specific tissue proteases such as PAR1, PAR4 [53], and many others. 

Our findings of differential granule release support and provide a mechanistic explanation for earlier studies examining the trophic effect of platelets on other cells. Independently, other groups have documented the differential release of alpha-granule proteins from platelets [61,62] and many have documented the presence of angiogenesis related proteins in alpha–granules. A morphometric evaluation of the platelet release reaction during thrombogenesis has demonstrated that platelets do not release all of their granules when they are incorporated into a thrombus, and retain the content of alpha-granules [63]. Thus the angiogenesis regulator-rich thrombus creates angiogenesis regulator-rich matrixes that can positively or negatively regulate angiogenesis within a narrow local milieu. It is also likely that other cell types containing secretory granules segregate angiogenic regulatory proteins to regulate differential release. For example, Weibel Palade bodies, the specific secretory organelles of endothelial cells, also contain several angiogenesis regulators and have been recently shown to differentially package and release P-selectin and VWF through the activity of tissue resident protease-activated receptors [64].

The role of platelets in the initiation, maintenance, modulation and inhibition of angiogenesis is becoming more appreciated even though the exact molecular mechanisms that regulate these processes remain somewhat mysterious. The differential packaging of specific proteins into alpha-granules is a consequence of at least two processes. Some proteins are synthesized by megakaryocytes and actively packed into the emerging platelet, others endocytosed while in circulation. The formation of alpha-granules is poorly understood, and is thought to involve recently described multivesicular bodies [65]. While an attractive target for future modulation of platelet content, the process has not been sufficiently understood. On the other hand, our findings of distinct populations of alpha-granules that can be differentially released suggest implications and potential for a substantial role in antiangiogenic therapy. It is now well accepted that the growth of a tumor beyond approximately 1 mm is dependent on the development of neovasculature [39]. The escape from dormancy and the related angiogenic switch engages the ability of platelets to promote new blood vessel growth by manipulating the protease activated receptors on platelets and triggering the selective release of predominantly proangiogenic factors. We [53] and others [61,62,66,67] have documented that the release of VEGF and endostatin is regulated by different thrombin receptors. One of the earliest descriptions of a constitutive and thrombin regulated platelet release of VEGF has been a report of Mohle *et al*. [68]. Other laboratories advanced this finding further, and showed that this release may be sequential. Early in wound healing or tissue injury, when thrombin levels are low, the high affinity thrombin receptor (Protease activated receptor 1, PAR-1) is activated, resulting in the release of VEGF [53,61,66,67]. As the concentration of thrombin increases, the low-affinity receptor PAR-4 is activated as well, resulting in the release of endostatin. This is, most likely, a very simplified picture of the complex reciprocal interactions that occur within the local microenvironment, but the principles explain, for the first time how angiogenesis could be stimulated in early tissue injury, and inhibited with gradual accumulation of scar tissue and thrombin. In a manner typical for tumor/host interactions a normal, physiological process is “hijacked” to enhance angiogenesis and thus exponential tumor growth. Because of the continuous tissue growth and expansion in most tumor settings, the overall effect of the platelet-releasate would be, on balance, proangiogenic [69,70,71].

There are two applications of this newly appreciated understanding about the role of platelets in tumor angiogenesis. For one, the protease-activated receptor-facilitated interaction between platelets and endothelial cells may be re-directed to maintain tumor dormancy through the action of biological response modifiers. Alternatively, the changes within the platelet angiogenesis proteome can be used to monitor cancer progression, therapeutic response or cancer screening. Both of these applications are likely to lead to significant improvements in cancer care, patients’ quality of life and the health care cost.

## 5. Platelet Protein Content as a Biomarker of Early Tumor Presence

Ever since the understanding that the process of angiogenesis represents a rate-limiting step in tumor progression [40], the need for circulating biomarkers was realized. In the subsequent 40 years, many laboratories investigated plasma and serum levels of the known angiogenesis regulators such as VEGF, bFGF, PDGF, ICAM, V-CAM, VE-Cadherin, endostatin, angiostatin, *etc*. [72,73,74,75,76,77]. The measurements proved to be very difficult. To examine the difficulties in measuring plasma VEGF for example: The half-life of VEGF in plasma is extremely short (seconds to minutes) and in order to have sustained levels of the protein, the levels need to be either very high or continuously replenished. This only happens in the case of very high tumor burden, or in terminal illness. Thus, plasma levels of VEGF may be by and large useful only for prognostication; if the levels are high, the patient is likely to do poorly. While helpful for prognostication and for identification of patients with disseminated disease, the usefulness of serum and plasma levels of VEGF, bFGF, or other angiogenesis regulatory proteins in early-stage tumors remained uncertain [78,79]. Despite 40 years of measuring plasma and serum levels of angiogenesis regulators and despite the numerous laboratories involved, any meaningful correlation of plasma and serum markers of angiogenesis with early tumor growth remains elusive. 

The second difficulty with the measurement of plasma VEGF is the miniscule amount of free circulating VEGF. Similarly to other very physiologically active circulating proteins, such as for example thyroxin, the levels of the free protein are carefully controlled, and remain in the picograms/mL range. In fact, sustained high levels of the protein are toxic [80], and the patient presents with a paraneoplastic syndrome. Furthermore, the physiological levels can have transient variations between 50–150 pg/mL—a three-fold change, increasing the variability between the individual measurements and decreasing the likelihood of meaningful statistical significance. Consequently, the numbers of patients required to power such a clinical study is often in the tens to hundreds of thousands. 

Third, ordinary physiological processes, such as acute inflammation, viral illness, or menstruation, may induce natural variations in plasma VEGF up to two-fold in either direction, further confounding the resolution of the measurements despite the sensitivity of ELISA. Finally, the physiological relevance of VEGF is as an acute responder and its spikes are quite transient. For example, after an initial induction of VEGF in acute hypoxia, and after the induction of the initial sprout, subsequent angiogenesis is sustained by other regulators such as bFGF, PDGF, angiopoietin 1, *etc*. The appreciation that the contents of serum may not reflect the contingent of platelet proteins was a very important milestone [5]. If the contents of serum does not reflect the full set of angiogenesis regulators in platelets [5] as had been presumed previously [72,75,81,82,83], then serum is not superior to plasma for this particular purpose. Since the majority of the relevant proteins remain associated with the clot [5,84], serum is only as good as plasma for measurement of angiogenesis regulators. 

There may not be a single biomarker that could inform on the angiogenic potential of a tumor. We proposed that the process of angiogenesis involves a sequential, spatially and temporally controlled, sequestration and release of angiogenesis regulators into and from platelets [53]. While this reciprocal interaction of platelets with the various tissue components may not be limited to tumor angiogenesis, it may be present in other physiological processes such as pulmonary hypertension [85], systemic sclerosis [86], or surgery [4,5]. However, our earlier study [4] indicates that, at least during surgery, the platelet response differs both in degree of protein sequestration in platelets as well as in the duration of this sequestration. The platelet angiogenic proteome of a mouse bearing dormant tumor contains numerous proteins, and the ratios of these proteins are distinctly different from those present in platelets of a mouse bearing angiogenic tumor [4,5], tumor of a different tissue of origin [4], or sham surgical wound.

## 6. Conclusions

In spite of the development of high-resolution imaging, and in spite of sensitive assays for detection of disseminated and circulating tumor cells, detecting clinically occult primary tumors or micrometastases still remains a major challenge. Tumors are currently being diagnosed with diameter that is usually larger than 0.5 cm and therefore they already contain over 10^9^ cancer cells [12]. These tumors can be referred to as being in a ‘late’ stage of tumor progression and may be shedding tumor cells into the circulation. The early detection of tumors that are likely to progress would be a crucial determinant of survival in patients with cancer, and have significant therapeutic implications. 

The clinically available diagnostic markers such as circulating cancer proteins (PSA, CEA or dopamine metabolites), circulating tumor cells or angiogenesis regulators, are only now being evaluated for early cancer diagnosis and none have proven sufficiently reliable. Our hope is that with further development of early platelet biomarkers and with identification of specific markers of dormancy, it may become possible to detect and treat cancer years before our patients become symptomatic, or even before the anatomical location of the cancer is feasible. However, crucial information such as the predictive markers for the fate of dormant tumors is still missing. Although several clinical trials are in progress to estimate risk of recurrence and prevalence of tumor dormancy [12], it is still impossible to predict which dormant tumors will eventually grow, and which will remain dormant. 

The ability of platelets to sequester markers related to cancer is a new discovery [4,5]. A successful implementation of the platelet angiogenesis proteome in the screening, monitoring and treatment of cancer will require basic and clinical studies to validate this new method. The ability to generate selective platelet releasates by manipulating protease-activated receptors may provide new opportunities for research and applications of tissue engineering and may aid in therapeutic strategies to promote or inhibit angiogenesis.

Since a shift in angiogenic potential is one of the hallmarks of tumors exiting the dormancy period, it is reasonable to assume that if platelets can selectively take up angiogenesis regulators in a manner that reflects the presence of tumors before the tumors are clinically evident, then analysis of the “platelet angiogenesis proteome” may be used for very early detection primary cancers, or cancer recurrence. We hope that the ability to detect clinically relevant cancer before a patient becomes symptomatic will provide a new avenue for early therapeutic intervention with novel agents. While the use of standard chemotherapy, radiation or surgery may not have been justifiable in the asymptomatic, clinically well patient with cancer, the recent emergence of non-toxic biological response modifiers holds a promise of “cancer without disease” [8], the unfinished work of angiogenesis pioneer Judah Folkman, M.D., cut short by his untimely death in January 2008. 

## References

[1] Almog N., Henke V., Flores L., Hlatky L., Kung A.L., Wright R.D., Berger R., Hutchinson L., Naumov G.N., Bender E., Akslen L.A., Achilles E.G., Folkman J. (2006). Prolonged dormancy of human liposarcoma is associated with impaired tumor angiogenesis. FASEB J..

[2] Naumov G.N., Bender E., Zurakowski D., Kang S.Y., Sampson D., Flynn E., Watnick R.S., Straume O., Akslen L.A., Folkman J., Almog N. (2006). A model of human tumor dormancy: an angiogenic switch from the nonangiogenic phenotype. J. Natl. Cancer Inst..

[3] Almog N., Ma L., Raychowdhury R., Schwager C., Erber R., Short S., Hlatky L., Vajkoczy P., Huber P.E., Folkman J., Abdollahi A. (2009). Transcriptional switch of dormant tumors to fast-growing angiogenic phenotype. Cancer Res..

[4] Cervi D., Yip T.T., Bhattacharya N., Podust V.N., Peterson J., Abou-Slaybi A., Naumov G.N., Bender E., Almog N., Italiano J.E., Folkman J., Klement G.L. (2008). Platelet-associated PF-4 as a biomarker of early tumor growth. Blood.

[5] Klement G.L., Yip T.T., Cassiola F., Kikuchi L., Cervi D., Podust V., Italiano J.E., Wheatley E., Abou-Slaybi A., Bender E., Almog N., Kieran M.W., Folkman J. (2009). Platelets actively sequester angiogenesis regulators. Blood.

[6] Black W.C., Welch H.G. (1993). Advances in diagnostic imaging and overestimations of disease prevalence and the benefits of therapy. N. Engl. J. Med..

[7] Brackstone M., Townson J.L., Chambers A.F. (2007). Tumour dormancy in breast cancer: an update. Breast Cancer Res..

[8] Folkman J., Kalluri R. (2004). Cancer without disease. Nature.

[9] Nielsen M., Thomsen J.L., Primdahl S., Dyreborg U., Andersen J.A. (1987). Breast cancer and atypia among young and middle-aged women: a study of 110 medicolegal autopsies. Br. J. Cancer.

[10] Hart I.R. (1999). Perspective: tumour spread--the problems of latency. J. Pathol..

[11] Harach H.R., Franssila K.O., Wasenius V.M. (1985). Occult papillary carcinoma of the thyroid. A "normal" finding in Finland. A systematic autopsy study. Cancer.

[12] Hedley B.D., Chambers A.F. (2009). Tumor dormancy and metastasis. Adv. Cancer Res..

[13] Wikman H., Vessella R., Pantel K. (2008). Cancer micrometastasis and tumour dormancy. APMIS.

[14] Retsky M., Demicheli R., Hrushesky W. (2001). Breast cancer screening for women aged 40–49 years: screening may not be the benign process usually thought. J. Natl. Cancer Inst..

[15] Baum M., Demicheli R., Hrushesky W., Retsky M. (2005). Does surgery unfavourably perturb the "natural history" of early breast cancer by accelerating the appearance of distant metastases?. Eur. J. Cancer.

[16] Demicheli R., Retsky M.W., Hrushesky W.J., Baum M. (2007). Tumor dormancy and surgery-driven interruption of dormancy in breast cancer: learning from failures. Nat. Clin. Pract. Oncol..

[17] Aguirre-Ghiso J.A. (2007). Models, mechanisms and clinical evidence for cancer dormancy. Nat. Rev. Cancer.

[18] Demicheli R., Terenziani M., Valagussa P., Moliterni A., Zambetti M., Bonadonna G. (1994). Local recurrences following mastectomy: support for the concept of tumor dormancy. J. Natl. Cancer Inst..

[19] Achilles E.G., Fernandez A., Allred E.N., Kisker O., Udagawa T., Beecken W.D., Flynn E., Folkman J. (2001). Heterogeneity of angiogenic activity in a human liposarcoma: a proposed mechanism for "no take" of human tumors in mice. J. Natl. Cancer Inst..

[20] Folkman J. (2004). Endogenous angiogenesis inhibitors. APMIS.

[21] Gimbrone M.A., Leapman S.B., Cotran R.S., Folkman J. (1972). Tumor dormancy *in vivo* by prevention of neovascularization. J. Exp. Med..

[22] Hahnfeldt P., Panigrahy D., Folkman J., Hlatky L. (1999). Tumor development under angiogenic signaling: a dynamical theory of tumor growth, treatment response, and postvascular dormancy. Cancer Res..

[23] Holmgren L., O'Reilly M.S., Folkman J. (1995). Dormancy of micrometastases: balanced proliferation and apoptosis in the presence of angiogenesis suppression. Nat. Med..

[24] Naumov G.N., Folkman J., Straume O. (2009). Tumor dormancy due to failure of angiogenesis: role of the microenvironment. Clin. Exp. Metastasis.

[25] O'Reilly M.S., Holmgren L., Chen C., Folkman J. (1996). Angiostatin induces and sustains dormancy of human primary tumors in mice. Nat. Med..

[26] Udagawa T., Fernandez A., Achilles E.G., Folkman J., D'Amato R.J. (2002). Persistence of microscopic human cancers in mice: alterations in the angiogenic balance accompanies loss of tumor dormancy. FASEB J..

[27] Indraccolo S., Favaro E., Amadori A. (2006). Dormant tumors awaken by a short-term angiogenic burst: the spike hypothesis. Cell Cycle.

[28] Aguirre-Ghiso J.A. (2006). The problem of cancer dormancy: understanding the basic mechanisms and identifying therapeutic opportunities. Cell Cycle.

[29] Barkan D., Kleinman H., Simmons J.L., Asmussen H., Kamaraju A.K., Hoenorhoff M.J., Liu Z.Y., Costes S.V., Cho E.H., Lockett S., Khanna C., Chambers A.F., Green J.E. (2008). Inhibition of metastatic outgrowth from single dormant tumor cells by targeting the cytoskeleton. Cancer Res..

[30] Uhr J.W., Marches R. (2001). Dormancy in a model of murine B cell lymphoma. Semin. Cancer Biol..

[31] Koebel C.M., Vermi W., Swann J.B., Zerafa N., Rodig S.J., Old L.J., Smyth M.J., Schreiber R.D. (2007). Adaptive immunity maintains occult cancer in an equilibrium state. Nature.

[32] Soucek L., Lawlor E.R., Soto D., Shchors K., Swigart L.B., Evan G.I. (2007). Mast cells are required for angiogenesis and macroscopic expansion of Myc-induced pancreatic islet tumors. Nat. Med..

[33] Quesnel B. (2008). Tumor dormancy and immunoescape. APMIS.

[34] Horak C.E., Lee J.H., Marshall J.C., Shreeve S.M., Steeg P.S. (2008). The role of metastasis suppressor genes in metastatic dormancy. APMIS.

[35] Chambers A.F. (2009). Influence of diet on metastasis and tumor dormancy. Clin. Exp. Metastasis.

[36] Gewirtz D.A. (2009). Autophagy, senescence and tumor dormancy in cancer therapy. Autophagy.

[37] Lu Z., Luo R.Z., Lu Y., Zhang X., Yu Q., Khare S., Kondo S., Kondo Y., Yu Y., Mills G.B., Liao W.S., Bast R.C. (2008). The tumor suppressor gene ARHI regulates autophagy and tumor dormancy in human ovarian cancer cells. J. Clin. Invest..

[38] Folkman J. (1995). Angiogenesis in cancer, vascular, rheumatoid and other disease. Nat. Med..

[39] Folkman J. (1992). The role of angiogenesis in tumor growth. Semin. Cancer Biol..

[40] Folkman J. (1971). Tumor angiogenesis: therapeutic implications. N. Engl. J. Med..

[41] Hanahan D., Folkman J. (1996). Patterns and emerging mechanisms of the angiogenic switch during tumorigenesis. Cell.

[42] Moserle L., Amadori A., Indraccolo S. (2009). The angiogenic switch: implications in the regulation of tumor dormancy. Curr. Mol. Med..

[43] Bayko L., Rak J., Man S., Bicknell R., Ferrara N., Kerbel R.S. (1998). The dormant *in vivo* phenotype of early stage primary human melanoma: termination by overexpression of vascular endothelial growth factor. Angiogenesis.

[44] Arbiser J.L., Moses M.A., Fernandez C.A., Ghiso N., Cao Y., Klauber N., Frank D., Brownlee M., Flynn E., Parangi S., Byers H.R., Folkman J. (1997). Oncogenic H-ras stimulates tumor angiogenesis by two distinct pathways. Proc. Natl. Acad. Sci. USA.

[45] Cao Y., O'Reilly M.S., Marshall B., Flynn E., Ji R.W., Folkman J. (1998). Expression of angiostatin cDNA in a murine fibrosarcoma suppresses primary tumor growth and produces long-term dormancy of metastases. J. Clin. Invest..

[46] Gilead A., Meir G., Neeman M. (2004). The role of angiogenesis, vascular maturation, regression and stroma infiltration in dormancy and growth of implanted MLS ovarian carcinoma spheroids. Int. J. Cancer.

[47] Tang Y., Wang M.T., Chen Y., Yang D., Che M., Honn K.V., Akers G.D., Johnson S.R., Nie D. (2009). Downregulation of vascular endothelial growth factor and induction of tumor dormancy by 15-lipoxygenase-2 in prostate cancer. Int. J. Cancer.

[48] Udagawa T. (2008). Tumor dormancy of primary and secondary cancers. APMIS.

[49] Watnick R.S., Cheng Y.N., Rangarajan A., Ince T.A., Weinberg R.A. (2003). Ras modulates Myc activity to repress thrombospondin-1 expression and increase tumor angiogenesis. Cancer Cell.

[50] Kang S.Y., Watnick R.S. (2008). Regulation of tumor dormancy as a function of tumor-mediated paracrine regulation of stromal Tsp-1 and VEGF expression. APMIS.

[51] Pietramaggiori G., Scherer S.S., Cervi D., Klement G., Orgill D.P. (2008). Tumors stimulate platelet delivery of angiogenic factors *in vivo*: an unexpected benefit. Am. J. Pathol..

[52] Handagama P., Rappolee D.A., Werb Z., Levin J., Bainton D.F. (1990). Platelet alpha-granule fibrinogen, albumin, and immunoglobulin G are not synthesized by rat and mouse megakaryocytes. J. Clin. Invest..

[53] Italiano J.E., Richardson J.L., Patel-Hett S., Battinelli E., Zaslavsky A., Short S., Ryeom S., Folkman J., Klement G.L. (2008). Angiogenesis is regulated by a novel mechanism: pro- and antiangiogenic proteins are organized into separate platelet alpha granules and differentially released. Blood.

[54] Pinedo H.M., Verheul H.M., D'Amato R.J., Folkman J. (1998). Involvement of platelets in tumour angiogenesis?. Lancet.

[55] Gasic G.J. (1984). Role of plasma, platelets, and endothelial cells in tumor metastasis. Cancer Metastasis Rev..

[56] Gasic G.J., Gasic T.B., Galanti N., Johnson T., Murphy S. (1973). Platelet-tumor-cell interactions in mice. The role of platelets in the spread of malignant disease. Int. J. Cancer.

[57] Nierodzik M.L., Karpatkin S. (2006). Thrombin induces tumor growth, metastasis, and angiogenesis: Evidence for a thrombin-regulated dormant tumor phenotype. Cancer Cell.

[58] Tang N., Tornatore P., Weinberger S.R. (2004). Current developments in SELDI affinity technology. Mass Spectrom. Rev..

[59] Seibert V., Wiesner A., Buschmann T., Meuer J. (2004). Surface-enhanced laser desorption ionization time-of-flight mass spectrometry (SELDI TOF-MS) and ProteinChip technology in proteomics research. Pathol. Res. Pract..

[60] Dvorak H.F. (1986). Tumors: wounds that do not heal. Similarities between tumor stroma generation and wound healing. N. Engl. J. Med..

[61] Ma L., Perini R., McKnight W., Dicay M., Klein A., Hollenberg M.D., Wallace J.L. (2005). Proteinase-activated receptors 1 and 4 counter-regulate endostatin and VEGF release from human platelets. Proc. Natl. Acad. Sci. USA.

[62] Ma L., Elliott S.N., Cirino G., Buret A., Ignarro L.J., Wallace J.L. (2001). Platelets modulate gastric ulcer healing: role of endostatin and vascular endothelial growth factor release. Proc. Natl. Acad. Sci. USA.

[63] Ashford T.P., Freiman D.G. (1968). Platelet aggregation at sites of minimal endothelial injury. An electron microscopic study. Am. J. Pathol..

[64] Cleator J.H., Zhu W.Q., Vaughan D.E., Hamm H.E. (2006). Differential regulation of endothelial exocytosis of P-selectin and von Willebrand factor by protease-activated receptors and cAMP. Blood.

[65] Heijnen H.F., Debili N., Vainchencker W., Breton-Gorius J., Geuze H.J., Sixma J.J. (1998). Multivesicular bodies are an intermediate stage in the formation of platelet alpha-granules. Blood.

[66] Ma L., Hollenberg M.D., Wallace J.L. (2001). Thrombin-induced platelet endostatin release is blocked by a proteinase activated receptor-4 (PAR4) antagonist. Br. J. Pharmacol..

[67] Perini R., Wallace J.L., Ma L. (2005). Roles of platelets and proteinase-activated receptors in gastric ulcer healing. Dig. Dis. Sci..

[68] Mohle R., Green D., Moore M.A., Nachman R.L., Rafii S. (1997). Constitutive production and thrombin-induced release of vascular endothelial growth factor by human megakaryocytes and platelets. Proc. Natl. Acad. Sci. USA.

[69] Brill A., Dashevsky O., Rivo J., Gozal Y., Varon D. (2005). Platelet-derived microparticles induce angiogenesis and stimulate post-ischemic revascularization. Cardiovasc Res..

[70] Brill A., Elinav H., Varon D. (2004). Differential role of platelet granular mediators in angiogenesis. Cardiovasc Res..

[71] Pintucci G., Froum S., Pinnell J., Mignatti P., Rafii S., Green D. (2002). Trophic effects of platelets on cultured endothelial cells are mediated by platelet-associated fibroblast growth factor-2 (FGF-2) and vascular endothelial growth factor (VEGF). Thromb. Haemost..

[72] Adams J., Carder P.J., Downey S., Forbes M.A., MacLennan K., Allgar V., Kaufman S., Hallam S., Bicknell R., Walker J.J., Cairnduff F., Selby P.J., Perren T.J., Lansdown M., Banks R.E. (2000). Vascular endothelial growth factor (VEGF) in breast cancer: comparison of plasma, serum, and tissue VEGF and microvessel density and effects of tamoxifen. Cancer Res..

[73] Fuhrmann-Benzakein E., Ma M.N., Rubbia-Brandt L., Mentha G., Ruefenacht D., Sappino A.P., Pepper M.S. (2000). Elevated levels of angiogenic cytokines in the plasma of cancer patients. Int. J. Cancer.

[74] George M.L., Eccles S.A., Tutton M.G., Abulafi A.M., Swift R.I. (2000). Correlation of plasma and serum vascular endothelial growth factor levels with platelet count in colorectal cancer: clinical evidence of platelet scavenging?. Clin. Cancer Res..

[75] Lee J.K., Hong Y.J., Han C.J., Hwang D.Y., Hong S.I. (2000). Clinical usefulness of serum and plasma vascular endothelial growth factor in cancer patients: which is the optimal specimen?. Int. J. Oncol..

[76] Nguyen M. (1997). Angiogenic factors as tumor markers. Invest. New Drugs.

[77] Wynendaele W., Derua R., Hoylaerts M.F., Pawinski A., Waelkens E., de Bruijn E.A., Paridaens R., Merlevede W., van Oosterom A.T. (1999). Vascular endothelial growth factor measured in platelet poor plasma allows optimal separation between cancer patients and volunteers: a key to study an angiogenic marker *in vivo*?. Ann. Oncol..

[78] Dosquet C., Coudert M.C., Lepage E., Cabane J., Richard F. (1997). Are angiogenic factors, cytokines, and soluble adhesion molecules prognostic factors in patients with renal cell carcinoma?. Cli. Cancer Res..

[79] Abendstein B., Daxenbichler G., Windbichler G., Zeimet A.G., Geurts A., Sweep F., Marth C. (2000). Predictive value of uPA, PAI-1, HER-2 and VEGF in the serum of ovarian cancer patients. Anticancer Res..

[80] Wong A.K., Alfert M., Castrillon D.H., Shen Q., Holash J., Yancopoulos G.D., Chin L. (2001). Excessive tumor-elaborated VEGF and its neutralization define a lethal paraneoplastic syndrome. Proc. Natl. Acad. Sci. USA.

[81] Gonzalez F.J., Rueda A., Sevilla I., Alonso L., Villarreal V., Torres E., Alba E. (2004). Shift in the balance between circulating thrombospondin-1 and vascular endothelial growth factor in cancer patients: relationship to platelet alpha-granule content and primary activation. Int. J. Biol. Markers.

[82] Spence G.M., Graham A.N., Mulholland K., McAllister I., Sloan J.M., Armstrong M.A., Campbell F.C., McGuigan J.A. (2002). Vascular endothelial growth factor levels in serum and plasma following esophageal cancer resection--relationship to platelet count. Int. J. Biol. Markers.

[83] Verheul H.M., Hoekman K., Luykx-de Bakker S., Eekman C.A., Folman C.C., Broxterman H.J., Pinedo H.M. (1997). Platelet: transporter of vascular endothelial growth factor. Clin. Cancer Res..

[84] Akerblom B., Lindahl T.L., Larsson A. (2002). ADP activation induces bFGF binding to platelets *in vitro*. Ups. J. Med. Sci..

[85] Eddahibi S., Humbert M., Sediame S., Chouaid C., Partovian C., Maitre B., Teiger E., Rideau D., Simonneau G., Sitbon O., Adnot S. (2000). Imbalance between platelet vascular endothelial growth factor and platelet-derived growth factor in pulmonary hypertension. Effect of prostacyclin therapy. Am. J. Respir. Crit. Care Med..

[86] Solanilla A., Villeneuve J., Auguste P., Hugues M., Alioum A., Lepreux S., Ducroix J.P., Duhaut P., Conri C., Viallard J.F., Nurden A.T., Constans J., Ripoche J. (2009). The transport of high amounts of vascular endothelial growth factor by blood platelets underlines their potential contribution in systemic sclerosis angiogenesis. Rheumatology (Oxford).

[87] Peterson J., Zurakowski D., Italiano J., Michel L., Fox L., Klement G., Folkman J. (2010). Normal ranges of angiogenesis regulatory proteins in human platelets. Am. J. Hematol..

